# Quantitative Assessment of Tip Effects in Single‐Molecule High‐Speed Atomic Force Microscopy Using DNA Origami Substrates

**DOI:** 10.1002/anie.202005884

**Published:** 2020-07-07

**Authors:** Charlotte Kielar, Siqi Zhu, Guido Grundmeier, Adrian Keller

**Affiliations:** ^1^ Technical and Macromolecular Chemistry Paderborn University Warburger Str. 100 33098 Paderborn Germany; ^2^ Present address: Institute of Resource Ecology Helmholtz-Zentrum Dresden-Rossendorf Bautzner Landstraße 400 01328 Dresden Germany

**Keywords:** biomolecular interaction, DNA origami, high-speed atomic force microscopy, protein–ligand binding, single-molecule studies

## Abstract

High‐speed atomic force microscopy (HS‐AFM) is widely employed in the investigation of dynamic biomolecular processes at a single‐molecule level. However, it remains an open and somewhat controversial question, how these processes are affected by the rapidly scanned AFM tip. While tip effects are commonly believed to be of minor importance in strongly binding systems, weaker interactions may significantly be disturbed. Herein, we quantitatively assess the role of tip effects in a strongly binding system using a DNA origami‐based single‐molecule assay. Despite its femtomolar dissociation constant, we find that HS‐AFM imaging can disrupt monodentate binding of streptavidin (SAv) to biotin (Bt) even under gentle scanning conditions. To a lesser extent, this is also observed for the much stronger bidentate SAv–Bt complex. The presented DNA origami‐based assay can be universally employed to quantify tip effects in strongly and weakly binding systems and to optimize the experimental settings for their reliable HS‐AFM imaging.

## Graphical abstract

**Heed the speed**: A DNA origami‐based assay for the quantitative evaluation of tip effects in high‐speed atomic force microscopy (HS‐AFM) is presented. Using streptavidin–biotin as a model system, it is shown that the rapidly scanned AFM tip may disrupt even strongly binding biomolecular complexes with dissociation constants in the femtomolar range.

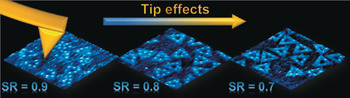

Atomic force microscopy (AFM) is a well‐established tool for the molecular‐level investigation of various biological structures and processes.^1^ While AFM provides high‐resolution images that may resolve even sub‐nanometer features, it is a notoriously slow technique with frame rates of the order of 0.1 min^−1^. With the introduction of high‐speed AFM (HS‐AFM), however, it became possible to visualize the dynamics of single biomolecules with a temporal resolution down to about 100 ms.^2^ Several studies have employed HS‐AFM for the real‐time investigation of selected biomolecular processes, including the walking of motor proteins^3^ and antibodies,^4^ enzyme reactions,^5^ and lipid bilayer dynamics.^6^ However, HS‐AFM is also increasingly employed in other fields of fundamental and applied research such as materials science^7^ and DNA nanotechnology.^8^

An important aspect in HS‐AFM studies is the effect that the rapidly scanned tip might have on the molecular dynamics. The comparatively high forces exerted by the tip may disrupt non‐covalent interactions between molecules and affect molecular motion. For systems characterized by relatively strong interactions such as actin–myosin or antibody–antigen complexes with dissociation constants (*K*_d_) in the pico‐ to nanomolar range,^9^, ^10^ such tip effects are generally considered to be of little importance.^11^ Weaker interactions, however, can be strongly disturbed by the exerted forces. Indeed, the assembly of SAS‐6 protein complexes with a *K*_d_ of circa 60 μm was effectively suppressed by such tip effects.^9^

To assess the influence of tip effects in HS‐AFM, it is usually suggested to carefully study the respective biomolecular system for a number of different scan parameters to determine the optimum setting that minimizes tip‐induced dissociation and desorption events.^11^ This is not only a rather time‐consuming task but also often quite challenging, in particular for strongly binding systems where the scanned tip may not completely disrupt the molecular interactions but only alter the observed dynamics and apparent equilibrium states. Such alterations can often be assessed only qualitatively and in a rather subjective manner. A more quantitative and standardized approach to assess the role of tip effects is needed to unambiguously rule out that the obtained results are tainted by any scanning‐induced artefacts.

Here, we utilize a DNA origami‐based single‐molecule assay to quantify tip effects in HS‐AFM. DNA origami decorated with single biotin (Bt) ligands and ligand pairs were exposed to the protein streptavidin (SAv). The SAv occupancy of each mono‐ and bidentate binding site on a number of selected DNA origami was continuously monitored over time by HS‐AFM in dependence of the employed line rate (LR) and set point ratio (SR). SAv–Bt is one of the strongest non‐covalently binding biomolecular systems with a dissociation constant in the femtomolar range^12^ and thus represents the best possible scenario, in which tip effects should play only a negligible role. However, as we show here, monodentate SAv‐Bt binding is notably disrupted by the scanned tip even under gentle scanning conditions. Although this effect is less pronounced for bidentate binding, tip effects are noticeable also in this much stronger system.

We employed Rothemund triangles^13^ featuring 9 Bt‐modified staples (see Figure 1 a). A single Bt modification is displayed in the center of each of the three trapezoids composing the triangular shape, while a Bt pair is displayed in one corner. The Bt modifications are attached to the staple strands via single‐stranded T_4_ spacers to ensure conformational freedom. Under these conditions, the two neighboring Bt modifications can each reach a corresponding binding pocket and thus facilitate bidentate binding of the tetrameric protein.^14^ The asymmetric arrangement of the Bt modifications enables the unambiguous discrimination of mono‐ and bidentate binding (see Figure 1 a).

**Figure 1:**
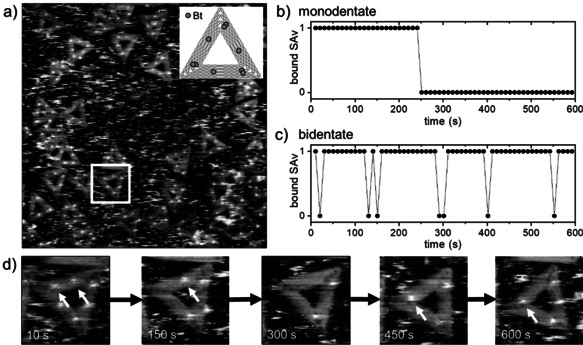
Mono‐ and bidentate SAv binding to Bt‐modified sites on a DNA origami during continuous HS‐AFM scanning. a) Overview HS‐AFM image (1×1 μm^2^) recorded at the beginning of the experiment. Inset: A schematic of the Bt modifications on the DNA origami, consisting of a single Bt in the center of each trapezoid and a Bt dimer in one edge. The occupancy of b) a monodentate and c) a bidentate binding site on the DNA origami indicated in (a) is determined from each AFM image and monitored continuously over a time course of 10 min. d) Selected zooms of the tracked DNA origami. The data in (b) and (c) were determined for the two binding sites located on the upper trapezoid. Bound SAv molecules at these sites are indicated by white arrows. The HS‐AFM images (1×1 μm^2^) were recorded at a free amplitude of 3.3 nm, SR=0.7, LR=50 Hz, and a resolution of 512×512 px^2^.

The DNA origami were immobilized on a mica surface and exposed to an excess of SAv. After incubation for at least 1 h to ensure binding equilibrium, the sample was continuously imaged by HS‐AFM over a time course of 10 min. Figure 1 b,c shows the SAv occupancy of a mono‐ and a bidentate binding site, respectively. Both binding sites are located on one trapezoid of the DNA origami indicated in Figure 1 a (see also Figure 1 d). As can be seen in Figure 1 b, the monodentate position carries a SAv molecule for the first 250 s, after which the protein dissociates from its ligand and leaves the site empty. The ligand site remains empty for the next 350 s. This indicates that after prolonged scanning, the bound protein is eventually being ripped off its ligand by the scanned tip, which subsequently prevents the binding of a free SAv molecule from solution. For bidentate SAv binding, the binding site is occupied by SAv molecules most of the time. This is due to the drastically lower *K*_d_ of the bidentate interaction.^14^ Nevertheless, the corresponding plot in Figure 1 c exhibits some fluctuations, indicating SAv dissociation and binding of free proteins from solution. This can directly be observed in the zoomed HS‐AFM images shown in Figure 1 d.

To obtain more meaningful data, we have monitored five DNA origami over the entire time course and analyzed the occupancy of all 30 SAv binding sites. The average binding yields for both binding modes are shown in Figure 2 as a function of time and for different LRs. In the initial images, DNA origami showing full occupancy were selected, so that all the plots start at an average yield of 100 %. For LR=10 Hz and 20 Hz, this initial value is more or less maintained over the entire 10 min. At LR=30 Hz, a small drop in the monodentate binding yield is observed after a few minutes. At LR=40 Hz, both the mono‐ and the bidentate yields show strong fluctuations almost over the entire 10 min. For LRs between 50 and 70 Hz, the bidentate binding yields stabilize rather quickly around 80 %. The monodentate yields, on the other hand, decrease more or less continuously over the first 200 s, after which they saturate at about 30 to 40 %. However, for LR=60 Hz and 70 Hz, the fluctuations of the monodentate binding yields in the saturation regime appear to be stronger than for 50 Hz and reach values below 20 % at several instances. Since image quality was continuously decreasing with increasing LR (see the Supporting Information, Figure S1), we were unable obtain meaningful images for LR>70 Hz.

**Figure 2:**
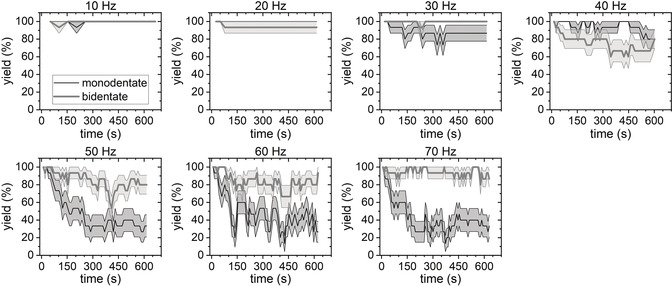
Average mono‐ and bidentate SAv–Bt binding yields determined from continuously recorded HS‐AFM images. Values represent averages of 15 individual binding sites with the shaded ranges corresponding to the standard deviations. The HS‐AFM images (1×1 μm^2^) were recorded at a free amplitude of 3.3 nm, SR=0.7, a resolution of 512×512 px^2^, and different LRs.

The results in Figure 2 clearly show that the fluctuations in SAv occupancy observed in Figure 1 are not just isolated events but occur to different degrees on all DNA origami. Furthermore, the obvious LR dependence suggests that it is indeed the rapidly scanned tip, which induces SAv‐Bt dissociation and prevents binding of free proteins from solution. In intermittent‐contact imaging, the impact of the cantilever tip onto the sample results in a vertical force pulse, which may reach values of several nanonewtons and thus affect the conformation and motion of proteins.^9^ Since the vertical impact force decreases monotonically as the SR increases from at least 0.5,^9^ we next evaluated the effect of different SRs on the determined SAv–Bt binding yields.

Figure 3 compares the time‐dependent mono‐ and bidentate binding yields at LR=50 Hz and different SRs. For monodentate SAv–Bt binding, increasing the SR from 0.7 to 0.8 and 0.9 indeed resulted in slight improvements, although the yields are still decreasing notably with imaging time and reach final values of about 50 and 65 %, respectively. For the bidentate case, the effect of the SR is less pronounced but still visible. This shows that the tip‐induced dissociation of the SAv–Bt complex indeed correlates with the impact force. However, even for SR=0.9, we still observe a strong reduction of the monodentate binding yield over only 5 min of continuous imaging. This is quite surprising considering the rather small free amplitude of only 3.3 nm and the exceptionally strong SAv–Bt interaction. It can be assumed that even higher SRs and/or smaller free amplitudes may result in further improvements. However, this will certainly result in lower image quality. This is already evident in the HS‐AFM images recorded at SR=0.9. As can be seen in Figure 4, SR=0.7 and 0.8 still yield HS‐AFM images of satisfying quality. For SR=0.9, however, image quality is visibly decreased. In particular, the individual SAv molecules on the DNA origami are no longer circular spots but exhibit tails along the fast‐scan direction, which is indicative of parachuting.^2^ We were not able to obtain usable images that allowed for a statistical evaluation at SR>0.9. This demonstrates that even for such a strongly binding system as SAv–Bt with a femtomolar *K*_d_ value, it may be very challenging to reduce tip effects to a negligible level simply by optimizing the imaging settings.

**Figure 3:**
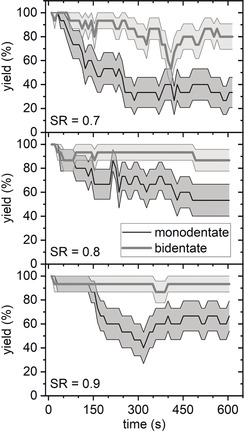
Average mono‐ and bidentate SAv‐Bt binding yields determined from continuously recorded HS‐AFM images. Values represent averages of 15 individual binding sites with the shaded ranges corresponding to the standard deviations. The HS‐AFM images (1×1 μm^2^) were recorded at a free amplitude of 3.3 nm, LR=50 Hz, a resolution of 512×512 px^2^, and different SRs.

**Figure 4:**
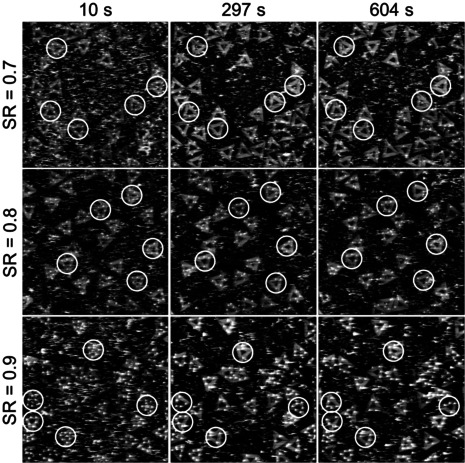
Selected HS‐AFM images (1×1 μm^2^) recorded at an amplitude of 3.3 nm, LR=50 Hz, a resolution of 512×512 px^2^, and three different SRs. White circles indicate the DNA origami used in the determination of the average binding yields shown in Figure 3.

In order to obtain a more consistent picture of the influence of SR and LR, we evaluated SR=0.8 and 0.9 also at other LRs (see Figures S4 and S5). To compare the different settings in a concise way, the average steady‐state binding yields in the final 100 s of the measurements have been determined and are presented in Figure 5. For all conditions evaluated, a decrease of the steady‐state binding yield can be observed with increasing LR. Due to the stronger interaction, the decrease for bidentate binding is less drastic than for monodentate binding. Nevertheless, also the bidentate binding yield decreases by 10 to 30 % for LR>30 Hz.

**Figure 5:**
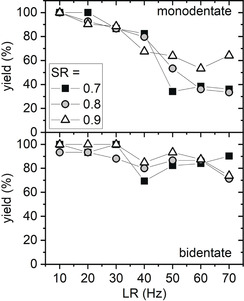
Steady‐state yields for mono‐ and bidentate SAv‐Bt binding in dependence of the LR for three different SRs. The steady‐state values were obtained by fitting a horizontal line to the average yields in the final 100 s interval of the respective time series (see Figure 2 and Figures S4 and S5 for the complete data sets). The uncertainties of the values as derived from the fits are smaller than the symbols. The HS‐AFM images (1×1 μm^2^) were recorded at a free amplitude of 3.3 nm and a resolution of 512×512 px^2^.

Concerning the influence of the applied SR, the situation is less clear. For bidentate SAv–Bt binding, the steady‐state yields do not show any obvious dependence on the applied SR but rather seem to scatter around a common average value. In the case of the monodentate binding, SR=0.7 and 0.8 mostly result in very similar steady‐state binding yields. Also SR=0.9 yields similar values as the other two SRs for LR ≤40 Hz. Only between 50 and 70 Hz, the steady‐state binding yields are noticeably higher at this SR. For example, at LR=70 Hz, steady‐state binding yields of around 35 % are obtained for SR=0.7 and 0.8. The corresponding value for SR=0.9 is increased to about 65 %.

These results are rather surprising, as higher SRs should lead to smaller vertical impact forces and thus fewer tip‐induced dissociation events. On the other hand, at a comparatively small free amplitude as used in the present experiments, the impact force does not scale as strongly with the applied SR as is the case for larger free amplitudes.^9^ This might provide an explanation for the observed weak or even absent dependence of the steady‐state binding yield on the SR. However, this weak dependence might also be an indication that the vertical impact force plays only a minor role in the investigated tip effects. In this case, the tip‐induced dissociation of the SAv–Bt complex would be dominated by lateral forces exerted on the proteins by the rapidly moving tip. These occur locally at the rising slopes of elevated surface features such as the SAv proteins attached to the DNA origami and are caused by the finite response time of the feedback loop. This interpretation would also be in line with the observed LR dependence.

Some of the data presented in Figures 2 and 3 and Figures S4 and S5 exhibit a rather peculiar behavior. Figure 2 reveals a strong decrease of the bidentate binding yield at LR=40 Hz. Further increasing the LR, however, results in a recovery of the yield, which for LR=70 Hz remains consistently above 80 %. Similarly, the monodentate binding yield obtained for SR=0.9 in Figure 3 initially decreases to about 40 %, but then recovers to a final and rather stable value around 60 %. This yield recovery may be caused by a direct interference of the tip as well. In particular, high lateral forces will remove a bound SAv by pulling it off its ligand. This will result in a horizontal pulling force acting also on the T_4_ spacer. The spacer will thus be stretched and lose its original random coil conformation. After rupture of the SAv–Bt bonds, the spacer will snap back and may assume a different conformation stabilized for example, by hydrogen bonding or electrostatic interactions. In this altered conformation, the Bt ligand may be partially shielded from the surrounding medium and thus display a lower SAv‐affinity. Subsequent continuous scanning, however, may result in the breakage of the stabilizing bonds and thus facilitate the spacer's return to its original conformation. This would cause the binding affinity to return to its original value, which may lead to a recovery of the binding yield. A similar phenomenon has previously been observed for DNA self‐assembled monolayers.^15^

Since the obtained monodentate binding yields in Figure 2 and Figures S4 and S5 mostly show an exponential decay with time, we also attempted to fit the data for LR≥30 Hz and extract tip‐induced dissociation rate constants (*k*_off,tip_ see the Supporting Information). The obtained *k*_off,tip_ values (Figure S9) are about 4 orders of magnitude larger than the *k*_off_ of SAv–Bt dissociation in bulk solution.^16^ Neglecting any tip‐induced alterations of *k*_on_, this drastic increase would manifest in an apparent *K*_d_ of the order of 100 pm. Somewhat surprisingly, however, the *k*_off,tip_ values do not show any clear trend, neither with regard to LR nor SR. This may be because *k*_off,tip_ does not characterize a single dissociation mechanism but rather a complex interplay of tip‐induced dissociation, equilibrium re‐binding, non‐equilibrium re‐binding in the presence of the scanning tip, and tip‐induced affinity variations as discussed above, all of which may exhibit different dependence on time, LR, and SR.

We have demonstrated the application of a DNA origami‐based single‐molecule assay for the quantitative evaluation of the effects that the rapidly scanned tip exerts on protein‐ligand complexes during HS‐AFM imaging. Such tip effects may disrupt biomolecular interactions, alter molecular motions, and thus lead to changes in the observed reaction and diffusion kinetics. It is generally assumed that tip effects play a dominant role only in weakly binding systems, whereas strongly binding ones are considered rather resilient.^9^ Nevertheless, in this work, we have employed one of the strongest non‐covalently binding systems, i.e., SAv–Bt, which has a dissociation constant in the femtomolar range. Bt ligands were immobilized on the DNA origami as single ligands and as ligand pairs to facilitate mono‐ and bidentate SAv binding, respectively. The SAv occupancy of the respective binding sites on selected DNA origami was monitored over a time course of 10 min by HS‐AFM in order to evaluate the influence of different scan parameters on tip‐induced SAv–Bt complex dissociation. Rather surprisingly, we observed that both mono‐ and bidentate SAv‐Bt complexes can be disrupted by the scanning tip even for a comparatively small free amplitude of only 3.3 nm. For SR=0.7 and monodentate SAv–Bt binding, the steady‐state binding yield after 500 s of continuous scanning decreased from about 100 % at LR=10 Hz to only about 35 % at 70 Hz. For SR=0.9, a steady‐state binding yield of 65 % was determined at LR=70 Hz. Although the scanning‐induced decrease in the binding yields was less pronounced for the bidentate complex with an even lower *K*_d_ value, it was still detectable.

Intriguingly, the presented results revealed a stronger influence of the LR on the tip‐induced SAv–Bt dissociation than of the SR. While this may to some extent be a result of the comparatively weak dependence of the vertical impact force on the SR at small free amplitudes, it may also hint at the importance of lateral forces. Comparing our results with equivalent experiments using the same assay but off‐resonance HS‐AFM imaging, which provides better control of the vertical impact forces,^9^ may shed some light on the relative contributions of both mechanisms.

Our results not only demonstrate that the rapidly scanned tip may induce significant artefacts in HS‐AFM investigations of strongly binding biomolecular systems but also highlight the importance of a careful and quantitative evaluation of the impact of the applied scan conditions on the biomolecular system. The assay presented here may serve as a valuable and versatile tool in this endeavor, not only because the DNA origami are straightforward to synthesize and easy to image but also because reliable quantitative information can be obtained from the analysis of only a handful of structures. Most importantly, however, numerous methods for the site‐specific decoration of DNA origami with a multitude of functional molecular species ranging from small molecules to large proteins and protein complexes have been reported in literature.^14^, ^17^ Therefore, we envision that this assay may be universally employed to quantify the sensitivity of various strongly as well as weakly binding biomolecular systems toward tip‐induced dissociation and to optimize the experimental settings to enable their reliable imaging by HS‐AFM with minimum disturbance.

## Conflict of interest

The authors declare no conflict of interest.

## Supporting information

As a service to our authors and readers, this journal provides supporting information supplied by the authors. Such materials are peer reviewed and may be re‐organized for online delivery, but are not copy‐edited or typeset. Technical support issues arising from supporting information (other than missing files) should be addressed to the authors.

Supplementary

Supplementary

Supplementary

Supplementary

Supplementary

Supplementary

Supplementary

Supplementary

Supplementary

Supplementary

Supplementary

Supplementary

Supplementary

Supplementary

Supplementary

Supplementary

Supplementary

Supplementary

Supplementary

Supplementary

Supplementary
