# Supplementary material for: Quantitative Assessment of Tip Effects in Single‐Molecule High‐Speed Atomic Force Microscopy Using DNA Origami Substrates
Source: Angew Chem Int Ed Engl. 2020 Jul 7;59(34):14336–41. doi: 10.1002/anie.202005884 (PMC7496922; doi:10.1002/anie.202005884)
Supplement: Supplementary file 1 — Supplementary [file ANIE-59-14336-s001.pdf]

Angewandte Chemie
Eine Zeitschrift der Gesellschaft Deutscher Chemiker

Supporting Information

Quantitative Assessment of Tip Effects in Single-Molecule High-Speed Atomic Force Microscopy Using DNA Origami Substrates

*Charlotte Kielar, Siqi Zhu, Guido Grundmeier, and Adrian Keller**

anie_202005884_sm_miscellaneous_information.pdf
anie_202005884_sm_Movie1.avi
anie_202005884_sm_Movie10.avi
anie_202005884_sm_Movie11.avi
anie_202005884_sm_Movie12.avi
anie_202005884_sm_Movie13.avi
anie_202005884_sm_Movie14.avi
anie_202005884_sm_Movie15.avi
anie_202005884_sm_Movie16.avi
anie_202005884_sm_Movie17.avi
anie_202005884_sm_Movie18.avi
anie_202005884_sm_Movie19.avi
anie_202005884_sm_Movie2.avi
anie_202005884_sm_Movie20.avi
anie_202005884_sm_Movie3.avi
anie_202005884_sm_Movie4.avi
anie_202005884_sm_Movie5.avi
anie_202005884_sm_Movie6.avi
anie_202005884_sm_Movie7.avi
anie_202005884_sm_Movie8.avi
anie_202005884_sm_Movie9.avi

Experimental Procedures

DNA origami assembly

Rothemund triangles[1] have been assembled from 208 staple strands (Metabion) and the M13mp18 scaffold as previously described[2] in 1 x TAE (Roth) containing 10 mM $MgCl_2$ (Sigma-Aldrich). The Bt-modified staple strands (Metabion, see Table S1) were added in 10-fold excess to the unmodified staple strands. Hybridization was carried out in a Thermocycler Primus 25 advanced (PEQLAB) by heating to 80°C and subsequent cooling to room temperature over a time course of 90 min. The samples were purified with 1 x TAE/$MgCl_2$ buffer by spin filtering using 100 kDa Ultra-0.5 ml centrifugal filters (Amicon). The concentration of the purified DNA origami solution was determined with an IMPLEN nanophotometer and adjusted with 1 x TAE/$MgCl_2$ to 5 nM.

Table S1. Sequences of all Bt-modified staple strands. The T_4 spacers indicated in bold face. Rothemund's original notation is used to identify the staples.

Modified staples	Oligonucleotide sequences 5' → 3'
t-1s6e	Bt-**TTTT**TTAGTATCGCCAACGCTCAACAGTCGGCTGTC
t-1s16e	Bt-**TTTT**ATTCGGTCTGCGGGATCGTCACCCGAAATCCG
t-1s26e	Bt-**TTTT**GCCAGTGCGATCCCCGGGTACCGAGTTTTTCT
t6s5g	Bt-**TTTT**CAGAGCCAGGAGGTTGAGGCAGGTAACAGTGCCCG
t6s15g	Bt-**TTTT**ATAAAGCCTTTGCGGGAGAAGCCTGGAGAGGGTAG
t6s25g	Bt-**TTTT**TCAATAGATATTAAATCCTTTGCCGGTTAGAACCT
t6s7f	ATTAAAGGCCGTAATCAGTAGCGAGCCACCCT**TTTT**-Bt
t6s17f	TAAGAGGTCAATTCTGCGAACGAGATTAAGCA**TTTT**-Bt
t6s27f	CAATATTTGCCTGCAACAGTGCCATAGAGCCG**TTTT**-Bt

Sample preparation for HS-AFM measurement

20 µl DNA origami solution with a concentration of 5 nM was pipetted onto a freshly cleaved mica substrate (1 cm diameter) mounted in a liquid cell and incubated for 2 minutes. Then, the substrate was washed with 1 ml of 1 x TAE/$MgCl_2$ buffer (pH 7.5) to remove unbound DNA origami. The liquid cell was then filled with 1 ml of 1 x TAE/$MgCl_2$ buffer (pH 7.5) containing 20 nM SAv (Sigma-Aldrich). After 1 h of incubation, the sample was subjected to HS-AFM imaging.

HS-AFM imaging

HS-AFM imaging was performed using a JPK Nanowizard ULTRA Speed using USC-F0.3-k0.3 cantilevers (f = 300 kHz, k = 0.3 N/m, NanoWorld). The images were recorded with scan sizes of 1 x 1 μm^2 and a resolution of 512 x 512 px^2. A constant free amplitude of 3.3 nm was used throughout the experiments.

Determination of binding yields from the recorded HS-AFM images

Time-dependent binding yields were determined by manually counting the occupation all the binding sites of five selected DNA origami in each recorded frame, averaging over a total of 15 monodentate and 15 bidentate SAv-Bt binding sites. The steady-state binding yields presented in Figure 5 have been determined by performing a linear fit with slope zero in the final 100 s (from 500 s to 600 s) of the saturation regime.

Additional Data

Selected AFM images of the different time series

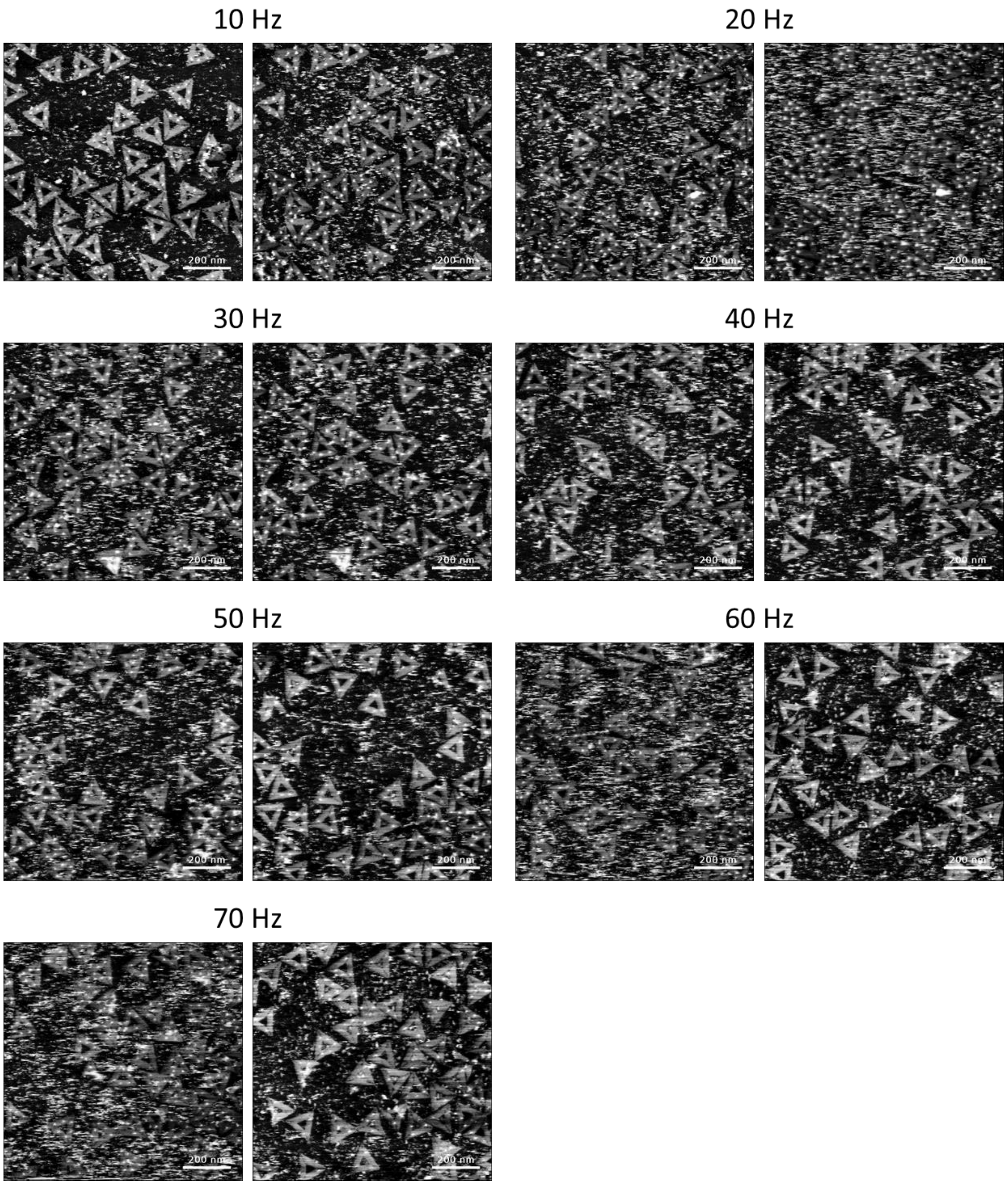

Figure S1. First (left) and last (right) AFM images recorded at the beginning and the end of the time series, respectively, for SR = 0.7 and different LRs.

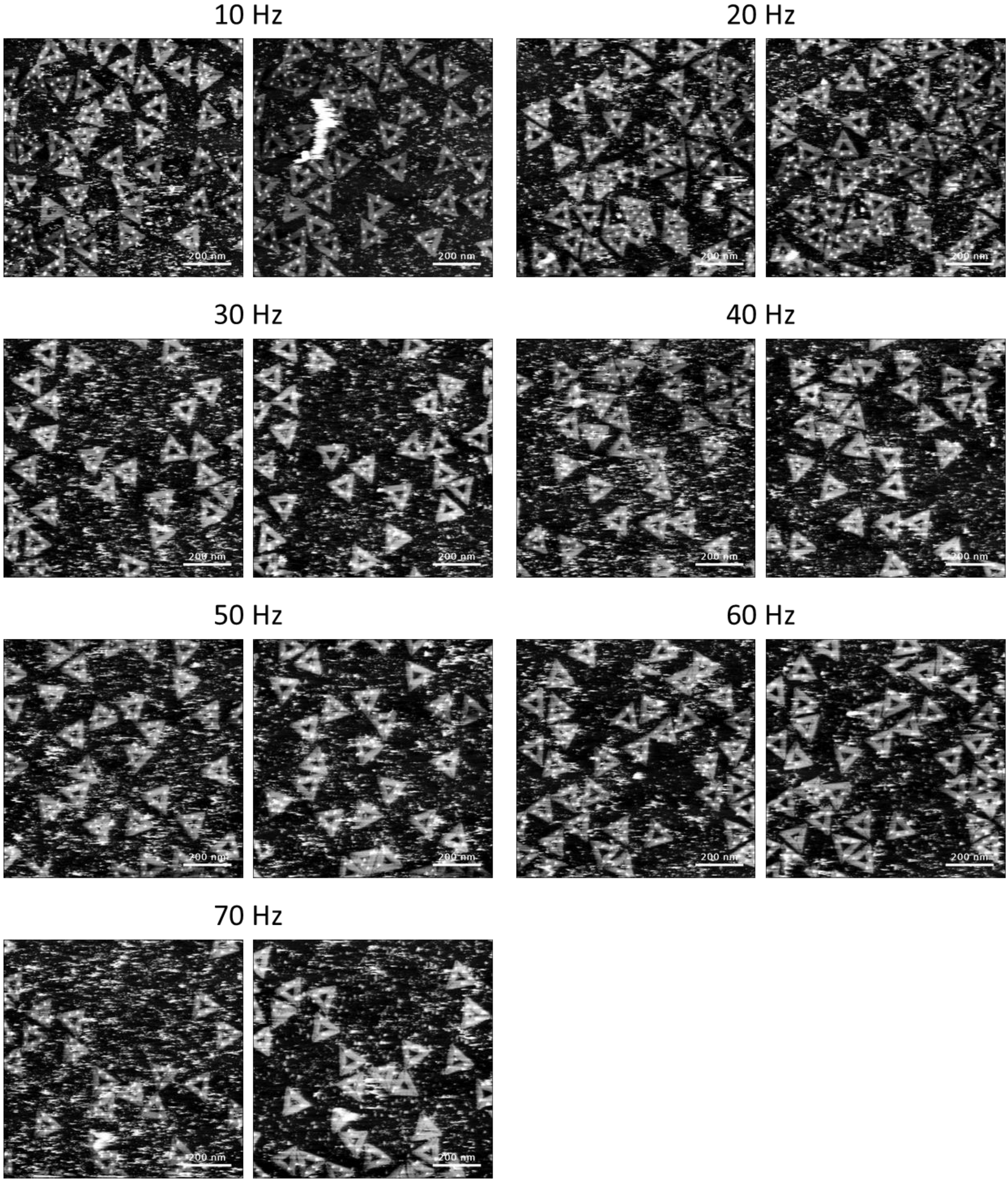

Figure S2. First (left) and last (right) AFM images recorded at the beginning and the end of the time series, respectively, for SR = 0.8 and different LRs.

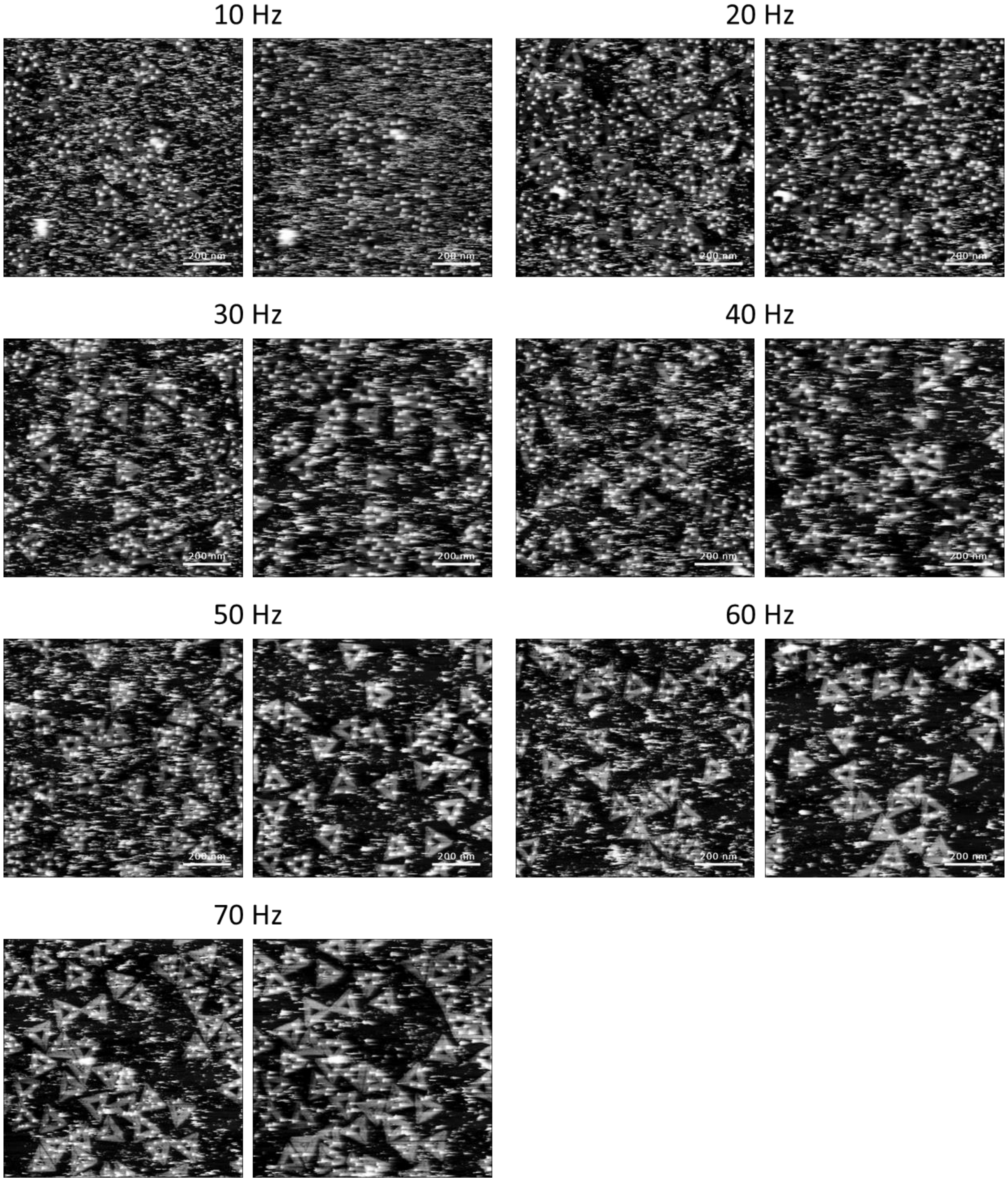

Figure S3. First (left) and last (right) AFM images recorded at the beginning and the end of the time series, respectively, for SR = 0.9 and different LRs.

Binding yields obtained at different LRs and SRs

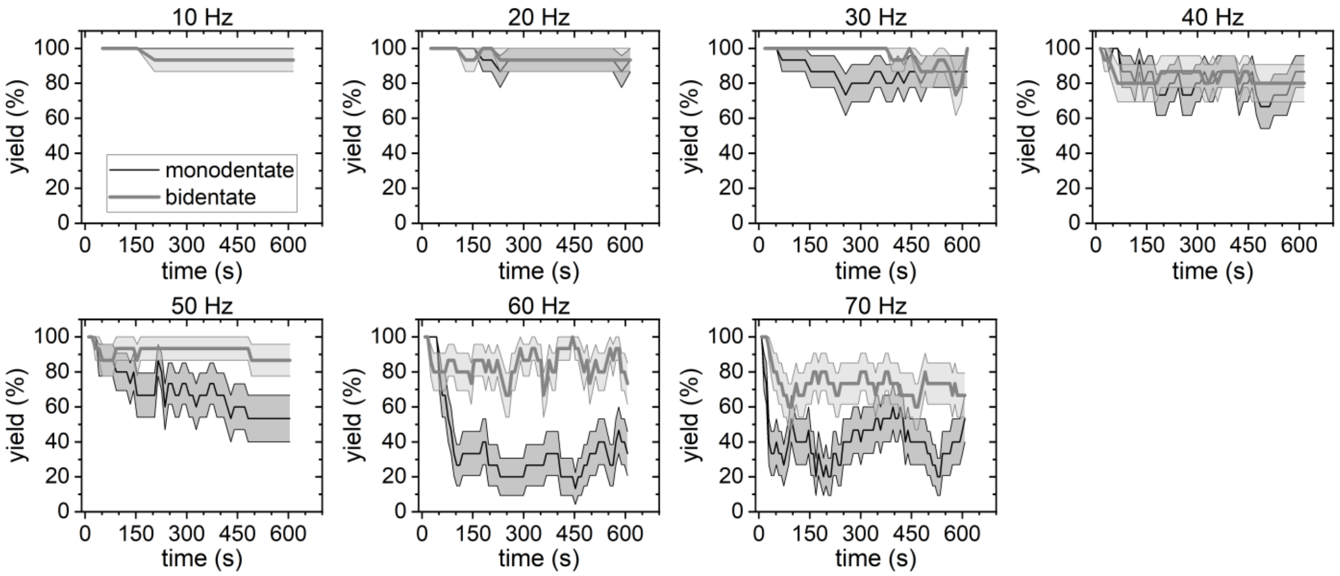

Figure S4. Binding yields obtained at different LRs and SR = 0.8.

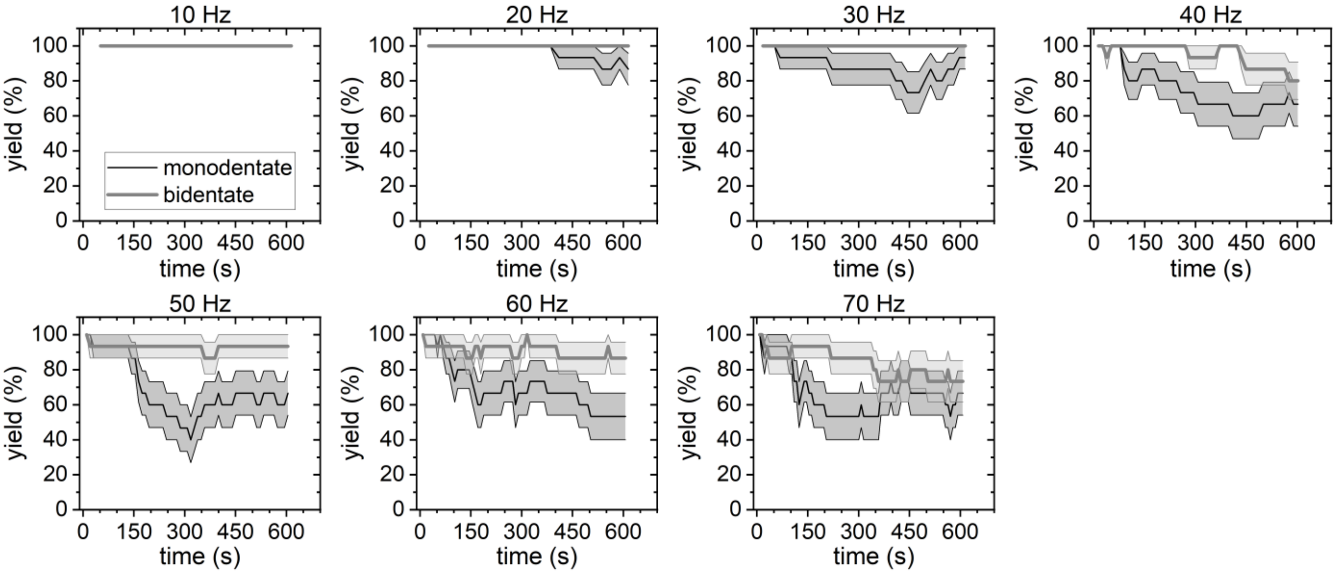

Figure S5. Binding yields obtained at different LRs and SR = 0.9.

Exponential decay fits of monodentate binding yields

The monodentate binding yields for all three SRs at LR ≥ 30 Hz have been analyzed by applying an exponential decay fit according to

$$\text{yield} = y_{\text{SS}} + (100\ \% - y_{SS})e^{-k_{\text{off,tip}}(t-t_0)}, \quad \text{(Equation 1)}$$

with the steady-state binding yield y_{SS} as given in Figure 5 of the main article, the time point t_0 at which the first HS-AFM image of the time series was recorded, and the dissociation rate constant $k_{\text{off,tip}}$. The fits are shown in Figures S6 to S8 and the obtained $k_{\text{off,tip}}$ values are presented in Figure S9.

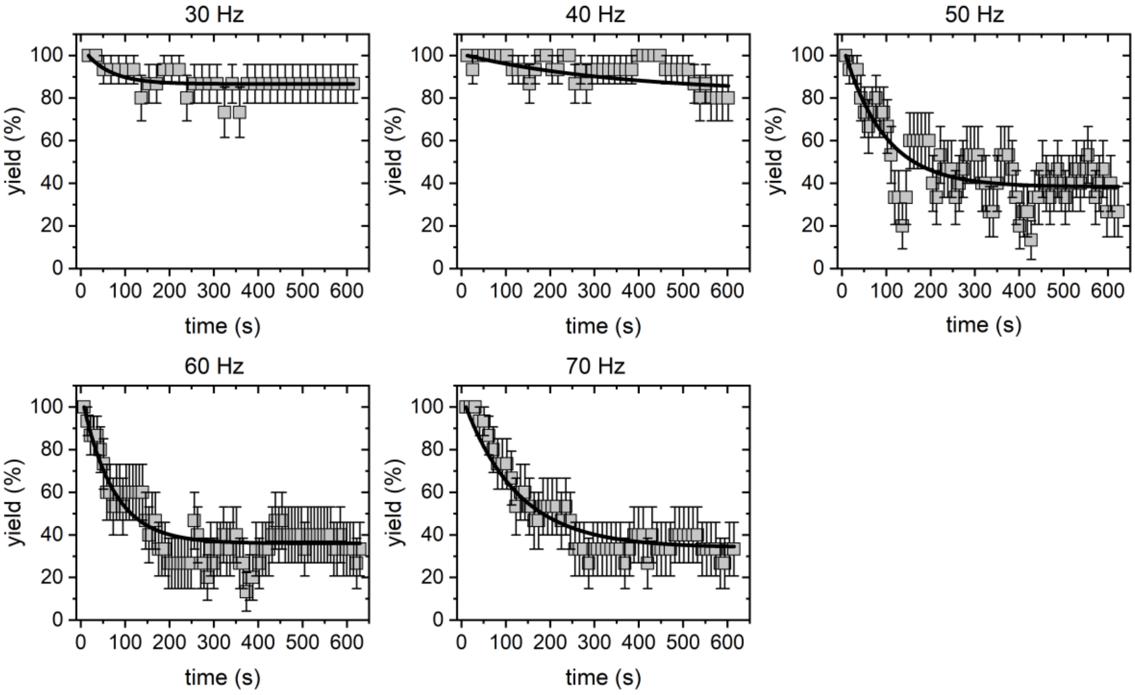

Figure S6. Monodentate binding yields obtained at different LRs and SR = 0.7 with corresponding fits according to equation 1.

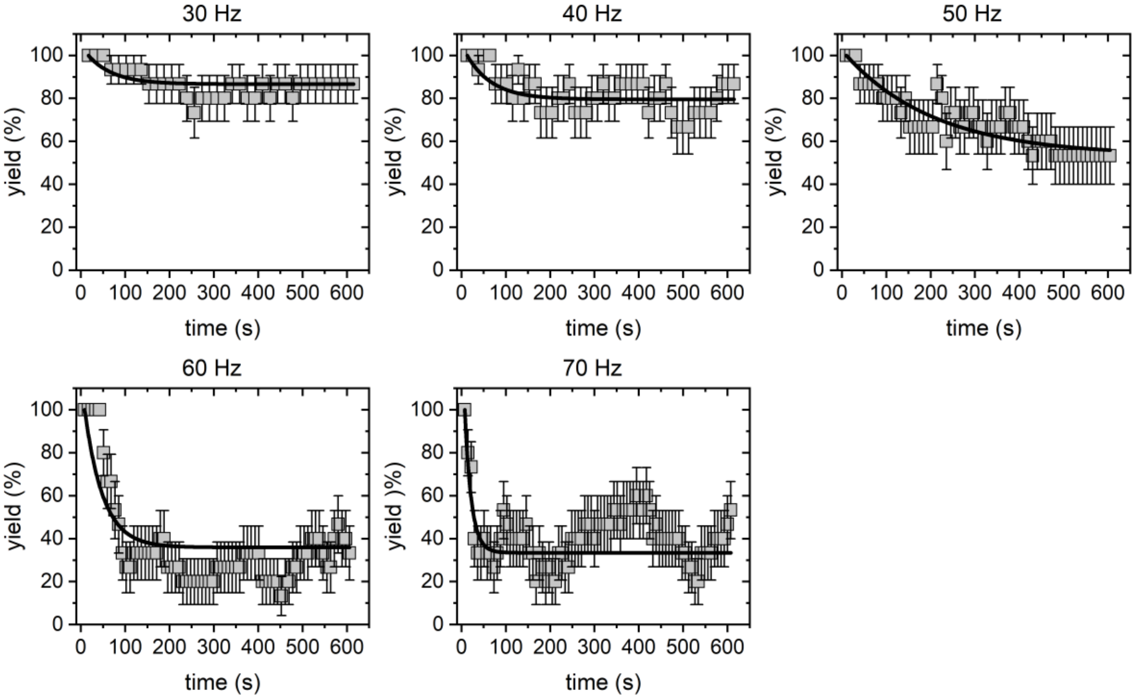

Figure S7. Monodentate binding yields obtained at different LRs and SR = 0.8 with corresponding fits according to equation 1.

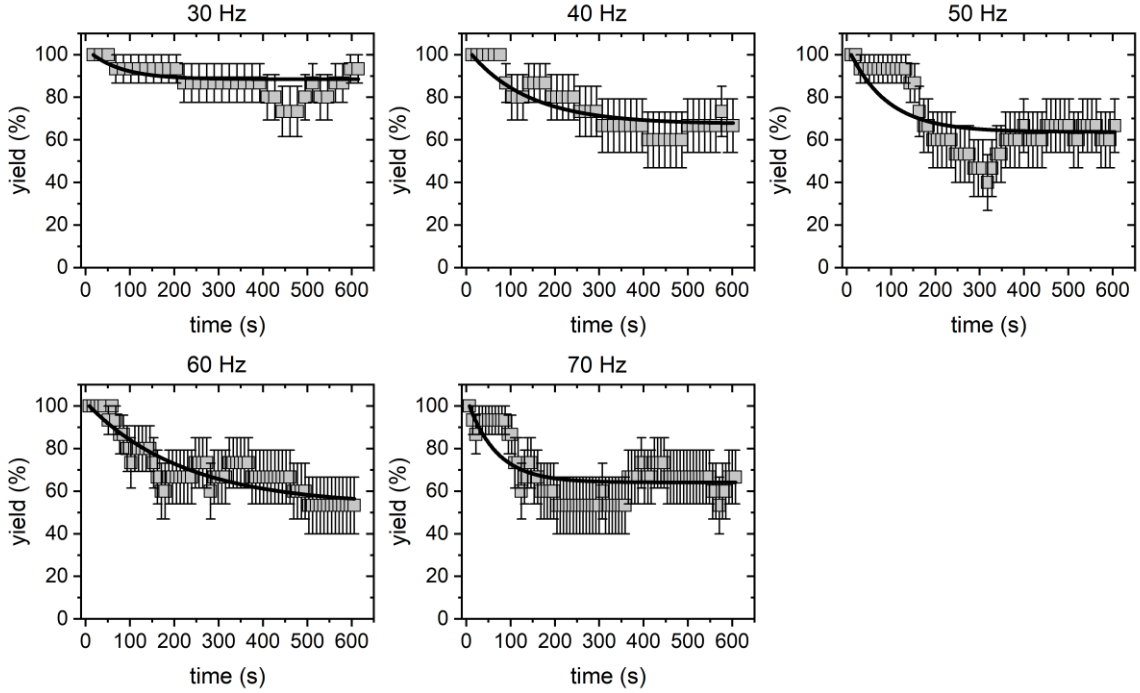

Figure S8. Monodentate binding yields obtained at different LRs and SR = 0.9 with corresponding fits according to equation 1.

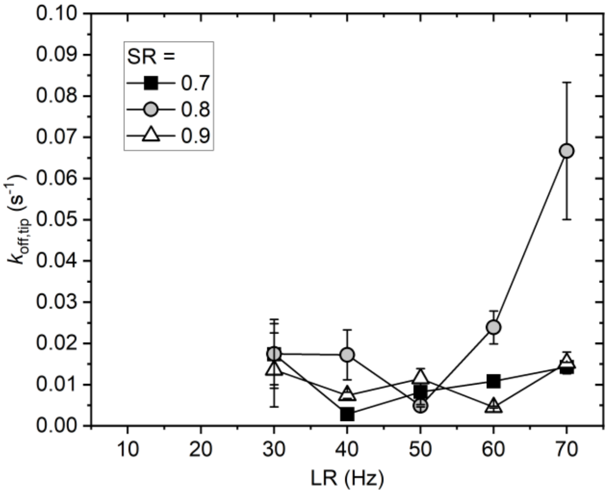

Figure S9. Tip-induced dissociation rate constants obtained from the fits shown in Figures S6 to S8. Note that the $k_{off,tip}$ values are about 4 magnitudes larger than the k_{off} previously obtained for SAv-Bt dissociation in bulk solution.[3]

References

[1] P. W. K. Rothemund, *Nature* **2006**, *440*, 297.
[2] C. Kielar, F. V. Reddavide, S. Tubbenhauer, M. Cui, X. Xu, G. Grundmeier, Y. Zhang, A. Keller, *Angew. Chem. Int. Ed. Engl.* **2018**, *57*, 14873.
[3] U. Piran, W. J. Riordan, *J. Immunol. Methods* **1990**, *133*, 141.

Author Contributions

Charlotte Kielar
Conceptualization: Equal, Data curation: Equal, Formal analysis: Lead, Investigation: Lead, Methodology: Equal, Validation: Equal, Visualization: Lead, Writing – original draft: Lead, Writing – review & editing: Supporting

Siqi Zhu
Data curation: Supporting, Formal analysis: Supporting, Investigation: Supporting, Validation: Supporting, Visualization: Supporting

Guido Grundmeier
Supervision: Supporting, Writing – original draft: Supporting, Writing – review & editing: Supporting

Adrian Keller
Conceptualization: Equal, Data curation: Equal, Formal analysis: Supporting, Methodology: Equal, Supervision: Lead, Validation: Equal, Writing – original draft: Supporting, Writing – review & editing: Lead
